# Real-World Intake of Dietary Sugars Is Associated with Reduced Cortisol Reactivity Following an Acute Physiological Stressor

**DOI:** 10.3390/nu15010209

**Published:** 2023-01-01

**Authors:** Nicola Di Polito, Anthea A. Stylianakis, Rick Richardson, Kathryn D. Baker

**Affiliations:** 1 School of Psychology, UNSW Sydney, Sydney, NSW 2052, Australia; 2 Department of General Psychology, University of Padova, Via Venezia 8, 35131 Padova, Italy

**Keywords:** cortisol, diet, sugar, fat, cold pressor test

## Abstract

There is increasing academic and clinical interest in understanding the nature of the relation between diet and response to stress exposure as a risk factor for mental illness. Cross-species evidence shows that conditions of chronic and acute stress increase the intake of, and preference for, caloric-dense palatable foods, a phenomenon thought to be explained by the mitigating effects of comfort foods on the activity of the stress-response network. It is largely unknown whether and how real-world dietary intake of saturated fat and sugars impacts stress responsivity in humans. Therefore, here we examined whether real-world dietary intake of saturated fat and sugars predicted salivary cortisol reactivity following an acute physiological stressor. Multilevel modelling of four salivary cortisol measures collected up to 65 min after the stressor on 54 participants (18–49 years old) were analyzed using a quadratic growth curve model. Sugar intake significantly predicted a weaker cortisol response following the Cold Pressor Test (CPT) controlling for BMI and gender, revealing an inhibitory effect of caloric-dense diets on cortisol reactivity to stress. As the consumption of sugar rose individuals had lower post-stressor cortisol levels, a smaller rate of increase in cortisol 20 and 35 min after the CPT, a lower cortisol peak, and an overall weaker quadratic effect. These observations add to a growing body of evidence reporting suppressive effects of high-energy foods on stress-associated glucocorticoids reactivity and are consistent with the comfort food hypothesis, where people are seen as motivated to eat palatable foods to alleviate the detrimental repercussions of stressor exposure.

## 1. Introduction

Poor quality diets and mental illness are major contributors to disability and disease [1]. In 2019, poor nutrition was one of the top three leading causes of death [2] and mental illness was the second highest cause of years lost to disability [2]. Although diet quality influences health through diverse physiological effects [3], there is increasing recognition that the escalation in consumption of caloric-dense diets, rich in saturated fat and refined carbohydrates, is a major challenge for metabolic and mental health [3,4,5,6]. Cross-sectional research has identified that several mental illnesses (e.g., major depression, schizophrenia, bipolar disorder, and post-traumatic stress disorder) are associated with increased intake of calories [7] as well as high consumption of processed foods, sugars, and saturated fats [8,9,10]. A growing body of work is acknowledging the benefits of nutritional medicine in psychiatry to potentially reduce the onset and symptoms of various mental disorders [11,12], including depression and anxiety [13,14], with a healthy diet and other lifestyle factors such as regular exercise recommended in clinical guidelines for initial steps towards management of such disorders [15,16]. 

There is increasing academic and clinical interest in understanding the nature of the relation between diet and response to stress exposure as a risk factor for mental illness [17,18,19,20,21]. Stress is a risk factor for depressive and anxiety disorders [22,23,24] and some have suggested that diet partially mediates the relation between exposure to stress and these mental illnesses. In other words, individuals exposed to stress consume unhealthy foods which in turn may contribute to mental illness. Indeed, there is cross-species evidence supporting the idea that stress reduces diet quality. Several studies in humans [25,26,27,28,29,30], rodents [31,32], and monkeys [33,34] show that chronic or acute stress increases the intake of, and preference for, caloric-dense palatable foods. However, the findings of a large population-based study revealed that whilst stress exposure predicts later depression and anxiety, diet quality did not mediate that effect [21]. Given that chronic or acute stress increases intake of calorically dense food the question arises as to why this occurs. One possibility is that people are motivated to eat palatable foods during times of high stress because these foods dampen the physiological stress response following acute stress [35,36]. From this perspective, “comfort eating” may alleviate the detrimental effects of stressor exposure. 

Although a healthy diet is often advised to alleviate stress and stress-related pathologies [37,38], scientific and experimental evidence directly supporting such recommendations is remarkably tenuous. That is, some studies have reported high calorie diets, rich in sugars and fat, enhance stress reactivity, across species. For example, some studies show high-fat diets enhance the corticosterone (a primary glucocorticoid in rodents) response to acute stress in rodents, specifically reporting a slower return to baseline levels post-stressor [39,40,41,42]. Similar results were reported in a study comparing stress reactivity during periods when rhesus monkeys had simultaneous access to both low and high caloric foods relative to periods with only low caloric food. In that study, levels of cortisol (a primary glucocorticoid in primates and humans) immediately after acute stress were elevated when animals had access to, and preferentially consumed, high caloric food (rich in saturated fat and sugars) compared to when only low caloric food was available [34]. Other experimental work has demonstrated that acute ingestion of sugars elevates and prolongs the cortisol response to acute stress in humans [43,44,45]. Additionally, in humans, a diet high in saturated fats has been linked to exaggerated cardiovascular reactivity [46], with even a single high-fat meal being associated with a heightened cardiovascular response to stress [47]. 

In contrast, other studies have reported the opposite pattern, such that high-calorie diets reduce stress responses. For instance, rats given the option to voluntarily consume sucrose or high-fat food in addition to standard diets have reduced corticosterone responsiveness to acute stress [32,48,49], in contrast to the exaggerated responses when high-fat diets are the only food provided [39,40,41,42]. Whereas acute ingestion of sugars elevates and extends the cortisol response to acute stress in humans [43,44,45], more prolonged sucrose consumption (three times per day for two weeks) instead reduces cortisol responsiveness to stress [50]. These latter studies in rats and humans are consistent with the common perception that the consumption of palatable “comfort” foods is connected to a short-term inhibition of the stress response. Given the high prevalence of Western-type diets and their alleged link to glucocorticoid responsiveness, there is a clear need to better understand how unhealthy dietary macronutrients, particularly saturated fat and sugars, influence the stress response system. Such knowledge may ultimately help us understand how patterns of inter-individual variation in stress reactivity are connected to mental health.

A common approach for examining hypothalamic-pituitary-adrenal (HPA) axis reactivity is the Cold Pressor Test (CPT), a widely deployed laboratory procedure used in research with human participants to reliably induce strong sympathetic nervous system activation (i.e., activation of the HPA and sympathetic-adrenal-medullary axes; [51]). Cortisol secretion in response to a stressor is a critical biomarker of HPA axis reactivity whose defining feature is its ample and ubiquitous intra- as well as inter-individual variability [44,52,53]. A non-exhaustive list of factors influencing cortisol response to an acute stressor includes sex and sex steroid-related factors, age, pregnancy-related issues, genetic and epigenetic factors, lifestyle and behavioral variables, psychological factors and interventions, personality, chronic stress and burnout, psychopathology, and circadian rhythm [53,54,55]. Notably, whether moderators of HPA responsiveness are reputed to be possible confounds as opposed to variables of interest is entirely dependent upon the conceptualization of the study and the research question at hand. Therefore, investigating how dietary factors affect CPT responses may not only inform understanding of the interaction of diet and stress, but will also provide information relevant for the future design of robust and properly controlled psychological studies using this task.

In the present study, we examined whether real-world dietary intake of saturated fat and sugars predicted salivary cortisol reactivity following an acute physiological stressor. Daily macronutrient intake was estimated using a self-report measure designed to collect information about participants’ diet over the previous 6 months. Based on the aforementioned literature, it was hypothesized that intake of saturated fat and sugars would predict inter-individual variation in cortisol reactivity following the CPT. 

## 2. Materials and Methods

### 2.1. Participants 

Seventy-two participants who were university students and members of the community were recruited for the study through online research participant pools at The University of New South Wales (UNSW) Sydney. Participants received course credit or $30 reimbursement for participating in the study. As the present study was conducted as part of a larger study examining intrusive memories (reported in [56]), there are some exclusion criteria that are not relevant for the aims of this particular study. Participants were screened using the Depression, Anxiety, Stress Scale 21 (DASS-21; [57]) and those with elevated final scores in the “Extremely Severe” range (Depression: 28+; Anxiety: 20+; or Stress: 34+) did not continue with the study and were provided with contact details for free counselling and psychological services at the university. Additional exclusion criteria were no access to a smartphone or tablet in order to download the application necessary to monitor intrusive memories (this data was not analyzed in the current study), past participation in a study relating to emotional memory intrusions, and meeting criteria for an anxiety disorder, depression, or post-traumatic stress disorder. 

Eighteen participants out of 72 were excluded from the data analysis; eight subjects were not exposed to the Cold-Pressor Task (i.e., their arm was placed in tepid water [28–36 °C] instead of cold water as a control condition in the study on intrusive memories reported in [56], six had poor quality (e.g., contaminated or degraded) saliva samples, and four did not correctly complete the diet questionnaire. However, six subjects were retained in the sample to maximize the sample size even though they did not follow all the instructions (i.e., they did not refrain from exercising twenty-four hours before the study, eating one hour before the study, or drinking caffeine or alcohol three hours before the study, or were taking antidepressants or contraceptives [58,59,60,61]). The final sample consisted of 54 (34 female) participants. The mean age was 24.7 years (*SD* = 6.8, median: 23.9 [Min: 18–Max: 49]), and the mean Body Mass Index (BMI) was 23 kg/m^2^ (*SD* = 4.3, median: 22 [Min: 16.8–Max: 36.1]). The study was approved by the UNSW Human Research Ethics Committee (project HC17947). 

### 2.2. Measures

#### 2.2.1. Pre-Screening Questionnaires

Participants completed the DASS-21 and provided demographic information, including their age, gender, and whether they had exercised in the last twenty-four hours, eaten one hour before the session, and consumed caffeine or alcohol within three hours of the experiment. 

#### 2.2.2. Australian Eating Survey Food Frequency Questionnaire (AES FFQ)

Macronutrient intake was measured with the Australian Eating Survey Food Frequency Questionnaire (AES FFQ), a self-report measure designed to collect information about dietary intake over the participants’ previous 6 months that has been thoroughly evaluated for reliability and validity in Australian toddlers, children, and adults and has demonstrated acceptable accuracy for ranking nutrient intakes [62,63,64,65,66]. The AES FFQ is a 120-item self-report measure assessing daily consumption of fruit, vegetables, dairy products, sweetened beverages, and snack foods, as well as the type of bread and dairy products consumed. It is a semi-quantitative FFQ with a standard portion size provided for each food item and determined using the ‘natural’ serving size (such as a slice of bread) or derived from the Australian Bureau of Statistics (ABS) unpublished data from the 1995 National Nutrition Survey (NNS) for children, adolescents, and adults. An individual response for each food or food type is required, with frequency options varying for different foods. For example, the scoring for some food items ranged from ‘Never’ to ‘4 or more times per day’ whereas some beverage items ranged from ‘Never’ up to ‘7 or more glasses per day’. The AES FFQ also has 15 supplementary questions about age, health-related behaviors (such as frequency of consumption of breakfast, takeaway food, and vitamin supplements), and sedentary activities. 

In the current study, proportions of daily energy intake from saturated fats and sugars derived from the AES FFQ were used to predict the cortisol response to the CPT. While this questionnaire directly provides percentages for the proportion of energy coming from saturated fats (coded as Saturated Fats in our model), the reciprocal value for sugars was manually computed. The percentage of total daily energy coming from sugars (coded as Sugars) was calculated using the following formula: [(sugars (*g*) × 17) × 100]/Energy (*Kj*). Sugars (*g*) were multiplied by 17 since each gram of sugar contains approximately 17 *Kj* [67,68]. 

#### 2.2.3. Cold Pressor Test (CPT) 

Participants placed their dominant arm (specifically their hand, wrist, and a portion of their forearm) in cold water (0–4 °C) for three minutes. Participants who removed their arm before three minutes were asked to re-submerge their arm as soon as possible. 

#### 2.2.4. Saliva Sampling and Analysis

Levels of cortisol were measured using saliva samples at intervals before and after the CPT collected via the passive drool method. Participants were asked to spit freely into a saliva collection aid (Salimetrics 5016.02) until 1 mL of saliva was collected. Saliva samples were stored at −80 °C until assay. After thawing, the samples were centrifuged at 1500× *g* for 10 min before being assayed in duplicate for cortisol concentrations using a competitive salivary cortisol enzyme immunoassay kit (Salimetrics 1-3002) according to the manufacturer’s instructions. Integrated optical density for each sample was calculated using a microplate absorbance reader (Biorad iMark) at 450 nm. Inter assay % CV was 8.1, and the intra assay % CV was 4.16 (both acceptable % CVs; Salimetrics, 2020). Cortisol levels are reported in µg/dL. 

### 2.3. Procedure

The laboratory component of the study was conducted between 11 a.m. and 6 p.m. in order to control for diurnal variation in cortisol levels [69]. After providing informed consent participants were asked to drink a glass of water to clear their mouths of content that could contaminate their saliva samples. They then completed the DASS and demographic information. Approximately 10 min after drinking water, participants provided a saliva sample to estimate their baseline cortisol levels. Participants were then presented with 40 color photographs selected from the International Affective Picture System (part of the experimental component not analyzed in the current study) after which they immediately completed the CPT.

Twenty, thirty-five, and sixty-five minutes following the completion of the CPT participants provided saliva samples. In the intervals between saliva samples, participants completed the AES FFQ. Other self-reported measures which were not analyzed in the present report were also taken during this time (Childhood Traumatic Events Scale, Impact of Event Scale-Revised, survey on menstrual cycle for women). 

### 2.4. Statistical Analyses

To test the effects of dietary sugar and saturated fats on cortisol after the CPT, we used a multilevel model (MLM) performed using the *GAMLj* package [70] in jamovi (*v* 1.8.1) [71,72] employing Maximum Likelihood estimation and a Satterthwaite approximation for degrees of freedom [73]. The MLM accounted for the nested structure of the data set where repeated measurements of cortisol across four time points (Level 1) were nested within individuals (Level 2). This approach offered several additional advantages. First, the MLM permitted the inclusion of all participants in the analysis, independently of whether there were subjects with missing measurements [74]. In this data set there were five participants with one missing cortisol measure. Second, the time intervals between measurements were not equal, and conveniently, in MLMs measurements need not be equally spaced in time [75]. Third, as we expected cortisol levels to increase and subsequently decrease, it was important for MLMs to allow for analysis of both linear and nonlinear trends [76,77]. Fourth, MLMs model variability at the between as well as within cluster level simultaneously, opening up the possibility of studying these variance components separately [78]. Fifth, multilevel modelling, in contradistinction to most repeated measures variance analyses, enables researchers to estimate and explain individuals’ variability in slopes, an approach sometimes referred to as “cross-level interactions” or “slopes as outcome analysis”. This is because the slope (i.e., the rate of change in a dependent variable), can be modelled as level 1 relationship as well as an outcome to be potentially explained by a level 2 predictor [79,80]. 

The MLM analysis was conducted in four steps. Step 1 was an intercept-only model in which data was clustered by participant. The unconditional model was used to confirm the improvement in fit of the subsequent models. Moreover, it provided the grand mean of the outcome variable and the variance estimates at each level of analysis. In Step 2, Time, Time^2^, and random slopes were added to probe the average variability in the linear and quadratic effect. To predict participants’ cortisol response to the CPT, Gender, BMI, Sugars, and Saturated Fats were specified as fixed predictors in Step 3. Saturated Fats and Sugars coded the proportion of daily energy intake coming from saturated fats and sugars, respectively. As past research has demonstrated gender differences in cortisol reactivity, gender was included in the analysis [81,82]. BMI was also specified as a control variable since past studies show associations between dietary patterns and BMI scores [83,84,85]. 

In Step 4, to test our main substantive question, cross-level interactions between the measurement variable and Sugars/Saturated Fats were added to the model. Models were compared at each step using a Likelihood Ratio Test (LRT). Given the aim of comparing nested models with different fixed effects using a LRT, a Maximum Likelihood (ML) estimation method was employed. However, since Restricted Maximum Likelihood (REML) estimation provides more reliable and less biased estimates for small sample sizes [86,87,88,89] we verified that the results of the final model (Step 4) with ML did not substantially differ from the output of an identical model run with REML. We also estimated model parameters excluding six subjects reporting medication use or non-compliance on recent food/alcohol consumption and exercise. Finally, to further explore the interaction between Sugars and Time^2^ a simple effect analysis of the effect of Sugars for the four initial cortisol measurement points was conducted. For each step model specification in lme4 R package formulation is provided. 

The final model included varying intercepts and slopes (both linear and quadratic) clustered by participant; Time, Time^2^, BMI, Gender, Saturated Fats, and Sugars as fixed predictors; and cross-level interactions between Sugars ∗ Time, Sugars ∗ Time^2^, Saturated Fats ∗ Time, Saturated Fats ∗ Time^2^. To improve the interpretability of the coefficients, BMI, Sugars, and Saturated Fats were grand mean-centered. Gender was coded as a simple categorical variable (0 = Male; 1 = Female). The dependent variable was cortisol response, which was specified as a time varying factor. Time was coded following the original four points of measurements of 0 and 20, 35, and 65 min after the CPT. However, to avoid convergence problems and to have more interpretable coefficients, the variable of time was represented in 20 min units. Moreover, to help answer our specific substantive questions about cortisol reactivity, Time was centered around the second measurement as this was the first measure after the stressor [90,91]. Additionally, as Time^2^ was a product of a pre-existing variable in our model, centering reduced the collinearity between the linear and the quadratic term [92]. Therefore, the final coding for Time was −1, 0, 0.75, and 2.25. 

## 3. Results

**Figure 1:**
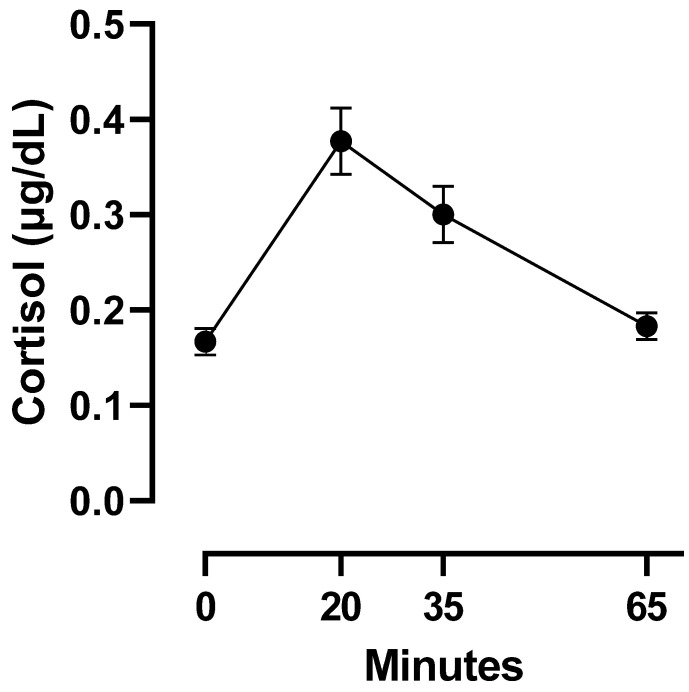
Mean (±SEM) cortisol depicted in minutes after the CPT. The baseline measurement before the CPT is depicted as “0” minutes.

The sample size, missing measures, means, standard deviations, and minimum and maximum scores for BMI, Saturated Fats, Sugars, and the four cortisol measurement points are reported in Table 1. The average cortisol levels at each measurement point with their respective standard errors are depicted in Figure 1. Intercorrelations between covariates were also estimated and are reported in Table 2.

*Step 1:* Cortisol ~ 1 + (1 | Participant)

In the intercept-only model the grand mean for cortisol response was 0.256 µg/dL (SE = 0.018, *t* (54.4) = 13.8, *p* < 0.001, 95% CI = [0.22, 0.29]). As expected, there was considerable variability in intercepts across clusters (var *=* 0.011, *SD* = 0.11). The intraclass correlation coefficient (ICC) was 0.278, indicating that, in the unconditional model, 27.8% of the variance in cortisol response was between participants, while the remaining variability was present within persons.

*Step 2:* Cortisol ~ 1 + Time + I(Time^2^) + (1 + Time + I(Time^2^) | Participant)

The random coefficient model showed a significant effect for Time (*B* = 0.082, *t* (53.9) = 4.7, *p* < 0.001, SE = 0.017, 95% CI = [0.05, 0.12]) and Time^2^ (*B* = −0.067, *t* (55.1) = −6.0, *p* < 0.001, SE = 0.011, 95% CI = [−0.09, −0.4]). The linear term represents the rate of change of the dependent variable at the second point of measurement (Time = 0), while the quadratic term describes how the linear effect of time changes through time (i.e., acceleration or deceleration) [90,91,93,94]. Therefore, at Time = 0 (the second measurement point) cortisol was increasing by 0.082 µg/dL per unit increase of Time, and that increase slowed by 0.067 µg/dL every 20 min. Random effect variances showed that subjects had different cortisol levels 20 min after the stressor (variability in intercepts) (var = 0.038, *SD* = 0.19). Moreover, there was substantial variability across participants’ linear (var = 0.011, *SD* = 0.10) and quadratic (var = 0.005, *SD* = 0.07) slopes, indicating considerable heterogeneity across subjects in the effect of Time and Time^2^.

Results of the LRT comparing the random-coefficient model to the intercept-only model demonstrate a significantly better fit for the random coefficient model (*χ*^2^ (5) = 141.8, *p* < 0.001). Additionally, a separate LRT for random slopes only showed that varying slopes for Time and Time^2^ significantly improved the fit of the model above and beyond the effect of the fixed predictors (*χ*^2^ (3) = 86.8, *p* < 0.001), confirming the presence of ample variability in slopes.

*Step 3:* Cortisol ~ 1 + Time + Gender + Saturated Fats + Sugars + BMI + I(Time^2^) + (1 + Time + I(Time^2^) | Participant)

In Step Three we added BMI, Gender, Sugars, and Saturated Fats as fixed predictors. No main effect was determined to be significant except for Time and Time^2^. Moreover, parameters included in both the conditional and random coefficients model, such as Time, Time^2^, and random variances, remained virtually unchanged from the model in Step 2. A LRT comparing the two models showed that the fit of the model was not improved by adding BMI, Gender, Sugars, and Saturated Fats as predictors (*χ*^2^ (4) = 1.9, *p* > 0.05).

*Step 4:* Cortisol~1 + Time + Gender + Saturated Fats + Sugars + BMI + I(Time^2^) + Time:Saturated Fats + Time:Sugars + I(Time^2^):Saturated Fats + I(Time^2^):Sugars + (1 + Time + I(Time^2^) | Participant)

In the fourth and final step of the analysis cross-level interactions between Sugars ∗ Time, Saturated Fats ∗ Time, Sugars ∗ Time^2^, and Saturated Fats ∗Time^2^ were added to the model. A visual inspection of the residuals’ histogram of the final model showed that albeit a slightly positive skewness being present, the errors of measurement are adequately approximating a normal distribution. Although recommendations vary, a favored rule of thumb is to consider data as substantially non-normal for values exceeding |2| for Skewness and |7| for Kurtosis [95,96,97]. Both measures of deviation from a normal distribution fall within the range of such recommendations for the residuals of the final model (1.3 for Skewness and 3.9 for Kurtosis).

A LRT comparing the fourth and third model approached significance (*χ*^2^ (4) = 8.16, *p* < 0.10). The fixed effect parameter estimates for the final model are reported in Table 3 and the random components in Table 4. Random effects variances show that after accounting for covariate effects there is still variability in intercepts (var = 0.03, *SD* = 0.18), linear slopes (var = 0.009, *SD* = 0.09), and quadratic effects (var = 0.004, *SD* = 0.06). Results show a significant effect for Time (*B* = 0.082, *t* (56.4) = 5.1, *p* < 0.001, SE = 0.016, 95% CI = [0.05, 0.11]) and Time^2^ (*B* = −0.067, *t* (55.5) = −6.5, *p* < 0.001, SE = 0.010, 95% CI = [−0.09, −0.5]). The linear term represents the rate of change in the outcome variable at Time = 0 (and thus at the second measurement point) holding covariates effects constant, while Time^2^ stands for how the effect of Time changes per unit change in Time, controlling for covariates effects [90,91,93,94]. Therefore, 20 min after the stressor cortisol was on average increasing by 0.082 µg/dL per unit of time, and that increase slowed down by 0.067 µg/dL each 20 min.

**Figure 2:**
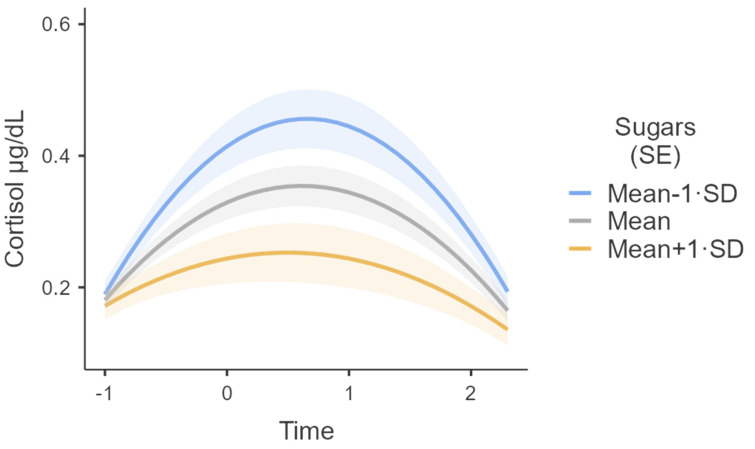
Variability in cortisol response (µg/dL) over time for High (+1 SD), Mean and Low (−1 SD) levels of sugar consumption. Semi-transparent areas indicate Standard Errors. Time was coded as −1, 0, 0.75, and 2.25 following the original four points of measurements of baseline and 20, 35, and 65 min after the CPT.

Sugars (*B* = −0.014, *t* (54.3) = −3.1, *p* = 0.003, SE = 0.005, 95% CI = [−0.02, −0.005]), Time ∗ Sugars (*B* = −0.008, *t* (56.2) = −2.8, *p* = 0.007, SE = 0.003, 95% CI = [−0.01, −0.002]) and Time^2^ ∗ Sugars (*B* = 0.005, *t* (55.3) = 2.9, *p* = 0.006, SE = 0.0018, 95% CI = [0.0016, 0.0088]) were also determined to be significant. The main effect of Sugars stands for the association between Sugars and cortisol at Time = 0, so that 20 min after the stressor subjects were predicted to have a cortisol response that is on average 0.014 µg/dL lower for every unit increase in Sugars. Moreover, Sugars significantly predicted both the linear and the quadratic slope. An increase in Sugars was associated with a smaller rate of increase at Time = 0 (the linear component), and a weaker quadratic effect [90,91,93,94]. Figure 2 illustrates how different levels of sugar consumption (i.e., low [−1 SD], average, and high [+1 SD]) predicted trajectories of cortisol response. Saturated fats were also determined to be negatively associated with cortisol secretion; however, neither the main effect or the interaction terms, both linear and quadratic, were statistically significant (*p* > 0.05).

We further explored the significant interaction between Sugars and Time with simple effects analyses of Sugars for different levels of Time (baseline and 20, 35, 65 min after the stressor). Results show no significant effect of Sugars at baseline (*B* = −0.0011, *t* (65.3) = −0.39, *p* = 0.701, SE = 0.002, 95% CI = [−0.007, 0.004]) or 65 min after the stressor (*B* = −0.005, *t* (57.8) = −1.8, *p* = 0.083, SE = 0.003, 95% CI = [−0.011, 7 × 10^−4^]). By contrast, there was a significant negative association of Sugars 20 min (*B* = −0.014, *t* (60.0) = −3.0, *p* = 0.004, SE = 0.005, 95% CI = [−0.024, −0.005]) and 35 min after the stressor (*B* = −0.017, *t* (59.2) = −3.0, *p* = 0.003, SE = 0.006, 95% CI = [−0.029, −0.006]). Thus, as the consumption of sugars increases, and while holding covariate effects constant, participants showed a weaker cortisol response for measures taken right after the stressor. More specifically, for each increase of one percentage point of total daily energy intake from sugars, 20 and 35 min after the stressor, the cortisol response was on average 0.014 µg/dL and 0.017 µg/dL lower, respectively. 

To verify whether tertiary factors (such as recent exercise, food consumption, and medication use) had a tangible impact on model parameters, the final model was also estimated excluding subjects (*n* = 6) reporting at least one of those factors. Finally, as REML has been shown to provide less biased estimates when dealing with small samples [86,87,88,89], we re-ran the step four model with REML (as opposed to ML) estimation. The results of those models were almost identical to those of the final model. 

## 4. Discussion

### 4.1. General Discussion

This study is a novel investigation of how real-world dietary intake of saturated fat and sugars influences the activity of the stress-response hormonal network in humans. Our data shows both saturated fat and sugars to be predictive of a weaker cortisol response to the CPT controlling for BMI and gender, revealing an inhibitory effect of caloric-dense diets on cortisol reactivity to stress. As the consumption of saturated fat and sugar rose, individuals had lower post-stressor cortisol levels, a smaller rate of increase in cortisol 20 and 35 min after the CPT, a lower cortisol peak, and an overall weaker quadratic effect. Although the effects of saturated fat and sugars were comparable, it was only sugar consumption that significantly (i.e., *p* < 0.05) predicted cortisol secretion following the CPT. Twenty minutes after the stressor individuals were predicted to show a cortisol response that was 0.014 µg/dL lower per increased percentage point of total daily energy coming from sugars. While the World Health Organization (WHO) strongly recommends keeping daily sugars intake to less than 10% of total energy [98], a cross-sectional study of over thirty thousand US children and adults found that sugars accounted for approximately 14% of total dietary energy [99]. Given that this estimate is around 4–5% higher than the advocated threshold, our data suggests that the average US citizen would experience a cortisol response to the CPT that is approximately 18% lower compared to someone adhering to WHO guidelines. Such pronounced dampening effects of dietary sugars on the stress response may carry important practical and clinical implications in managing stress and stress-related disorders.

The observations in the present study are at odds with some previous research reporting that calorically dense diets exacerbate stress-induced glucocorticoid hormones responsiveness [34,39,40,41,42,46,47]. In contrast to this literature, several studies, aligning with the results presented here, show that intake of carbohydrates and dietary sugars is associated with a decrement in HPA axis reactivity in both humans and rodents [32,48,49,50,100,101,102,103]. For instance, in humans, a single day of a carbohydrate- and sugar-rich diet has been shown to lower stress-provoked cortisol reactivity and reduce feelings of depression after a stress-inducing mental arithmetic task [103]; the consumption of sucrose (3 times per day for 2 weeks), but not of the artificial sweetener aspartame, has also been associated with an inhibitory effect on cortisol secretion following the Montreal Imaging Stress Task [50]. Similarly, glucocorticoid responsiveness following acute and repeated restraint is reduced in rodents freely choosing to eat lard and/or sugar as opposed to chow [32,48,49]. Being able to choose palatable high-energy foods is thought to be a critical feature of the effect, suggesting that control and discretion over one’s diet may be more important than just calories or food composition [48,104]. Our findings add to the extant literature examining short-term experimental manipulations of diet in humans by demonstrating that real-world sugar consumption predicts suppression of cortisol secretion following a physiological stressor.

In humans, stress generally increases desire to consume highly palatable foods relative to more nutritious alternatives [25,26,27,28,29,30], even for those people whose total caloric intake is diminished or in the absence of hunger [105,106]. This preference is particularly true for sugary foods, with a majority of individuals reporting elevated consumption of sweet foods specifically during times of high stress for self-reported reasons of psychological comfort and relief [28,105,107]. Indeed, there is convincing evidence showing that people experience improved mood, cheerfulness, and reduced feelings of stress and discomfort after ingestion of palatable calorically rich foods, and in particular sweet foods [108,109,110,111]. 

Despite comfort eating being an established and widespread eating pattern, the physiological underpinnings of this phenomenon are still largely unknown, especially in humans. One suggested mechanism purporting to explain how sugar intake dampens reactivity to stress stems from the metabolic-brain feedback model [31,50]. This model is built on research indicating that consumption of high-energy food, due to its anabolic properties, cues the brain to truncate stress-induced HPA axis activity [31,101,112]. Given that glucocorticoid secretion triggers catabolic processes needed to meet energy requirements under conditions of chronic and acute stress [113,114], this could plausibly serve as an adaptive mechanism by which release of steroid hormones is calibrated in relation to current energy availability [115]. In fact, previous research shows that glucocorticoid circulation aids homeostatic readjustment by increasing energy intake and prompting the formation of glycogen and fats [31]. Sugars may thus help provide the fuel necessary to match the metabolic demands of stress, which would, in turn, decrease the need for glucocorticoids-directed energy catabolism. Substantiating this conclusion, preclinical research shows that sucrose consumption suppresses HPA axis activity and restrains catabolism of bodily energy stores [32,101,116].

One putative mechanism by which sucrose consumption suppresses HPA axis activity is via an opioid-mediated suppression of corticotropin-releasing factor (CRF) secretion from hypothalamic neurons [49,101,102,116,117,118]. Another brain mechanism speculated to be involved in sugar-induced reductions of HPA axis reactivity to stress is via a strengthening of the tonic inhibition of the HPA axis by the hippocampus [50,119]. In humans, deactivation of the hippocampus is associated with higher circulating cortisol levels after stressor-exposure [120], suggesting that deactivation of this region disinhibits the HPA axis and initiates stress hormone release. Dietary sugar, however, appears to strengthen the inhibitory “brake” on the HPA axis. In support of this, an intervention involving two weeks of three times daily consumption of beverages sweetened with sucrose (but not the artificial sweetener aspartame) increased activity in the hippocampus (i.e., it prevented the deactivation effect) after a social stressor and reduced salivary cortisol in humans [50]. Therefore, long-term high sugar consumption may reduce HPA axis activation via alterations to hippocampal activity. 

Our findings, aligning with growing amounts of evidence across species, suggest that people may be motivated to eat increasing quantities of high-energy palatable foods because of these nutrients’ inhibitory effects on the stress-response system. Therefore, although comfort eating is almost invariably thought of as unambiguously harmful, prompting the development of stress-eating interventions to reduce such behavioral tendencies [121], the present work adds to the literature pointing toward short-term beneficial function of stress-induced eating of calorically dense foods. However, this cannot be considered a tenable long-term coping strategy as habitual consumption of such foods leads to intra-abdominal obesity [122], which is robustly associated with metabolic syndrome, hypertension, type 2 diabetes, cardiovascular disease, morbidity, and mortality in humans [123,124,125,126]. Although obesity is certainly a disease of multifactorial aetiology, research uncovering latent relations between diet, stress, and overconsumption of high-energy foods may help explain the rampant obesity epidemic present in our society. 

Understanding why people eat high-energy foods during periods of stress may also provide insight into coping mechanisms for depression and anxiety disorders. Preclinical studies demonstrate that consumption of palatable food dampens anxiety- and depressive-like behaviors in animals exposed to chronic or acute stressors [36,127]. In addition, as stronger depressive symptoms have been associated with increased comfort eating in humans [128], and depression is characterized by profound changes in appetite as well as excessive cortisol secretion, individuals suffering depression may be particularly susceptible to overeating of high-energy foods as a form of self-medication to deal with stressors [129]. Individuals with disordered eating syndromes may also overeat for similar reasons [31]. Moreover, it has been speculated that the overeating, and its reciprocal weight gain, often observed in people experiencing depression [130] could be partially explained by individuals experiencing less pleasure from calorically dense foods and overeating to compensate [31].

### 4.2. Limitations

A strength of our study is the examination of the effect of real-world dietary consumption of sugars on stress reactivity, complementing previous studies using experimental short-term food manipulations. Adding to the relevance of our results, the suppressive effects of sugar on cortisol secretion following an acute physiological stressor have been estimated controlling for two important potentially confounding factors, BMI and gender. Nevertheless, results presented here should be considered in light of several limitations. The main weakness of the present work is the low number of participants eligible to be included in the analysis. Although the total (already limited) sample consisted of 72 subjects, 18 of those had to be excluded, resulting in a suboptimally small and likely underpowered sample size. More than just potentially biasing overall results, such a small sample size precludes the inclusion in the analysis of other possible confounding variables to control for their influence on final model parameters. Furthermore, given that recruitment of prospective subjects was achieved through the online research participant pools at a university, sampling bias is conspicuously apparent, dramatically limiting the generalizability of these results. For instance, although participants’ ages ranged from 18 to 49, only five subjects were older than 35. Given that emerging adulthood is a risk period for poor mental health and diet quality it is surprising that the associations between diet, stress, and mental health are relatively underexplored compared to other stages of the lifespan [131]. The findings of the present study in people predominantly aged 18–35 may, therefore, increase knowledge of how diet influences stress responses in emerging adulthood.

Further constricting the generalizability of our findings is the fact that participants were instructed to refrain from engaging in activities that could possibly bias the cortisol response. While this enhances experimental control, it weakens our confidence in assuming that these results would adequately translate outside of controlled experimental conditions, where such behaviors are ubiquitous. In addition, the CPT, while being a well-validated laboratory procedure to induce HPA axis activation, is different from most stressors encountered in real-life situations, tempering conclusions around whether this effect would replicate under real-world circumstances.

Finally, it must also be emphasized that while variability in cortisol secretion has been putatively explained with a series of covariates, due to some uncontrolled extraneous variables, it is possible that untested confounding factors could be responsible for the estimated inhibitory effect of calorically dense foods on cortisol secretion.

### 4.3. Future Directions

Given that research regarding comfort eating is still in its early stages, there are many promising avenues of discovery to pursue. While convincing preclinical evidence documenting the stress-eating relationship exists, human subject research, although so far mostly consistent with results demonstrated in other species, is only just beginning. Future studies should thus aim to expand our understanding of this phenomenon in humans, starting with investigating whether the effects reported in the animal literature generalize to people. Importantly, studies with human participants conducted so far have focused almost exclusively on acute socially evaluative stressors presented in a laboratory environment. Although the present work provides novel insights into how sugar and saturated fat consumption is related to reactivity to a physiological stressor, research related to more diverse, ecologically valid, and naturally occurring stress manipulations is required. Another neglected area of investigation is how different kinds of stress (i.e., anticipated as opposed to unexpected and unpredictable, tractable as opposed to intractable, mild or severe, acute as opposed to chronic, etc.) relate to the stress-eating relation and its reciprocal inhibitory effects on the HPA axis. In addition to exploring how different types of stress, stressors, and emotions impact this effect in humans, future research should also prioritize examining physiological mechanisms linked to stress beyond cortisol. Past studies have almost invariably adopted activation of the HPA axis as the sole outcome measure of stress-responsivity, and yet, autonomic and immune system functioning are as importantly altered by stressor exposure.

It will also be important to uncover possible predisposing factors leading to susceptibility in reaching for high-energy tasty foods to improve one’s mood and to a stronger (or weaker) inhibition of the stress response. Finally, although progress in explicating how dietary sugars affect stress-induced glucocorticoid secretion is being made, the mechanisms underlying this relation are still speculative and remain poorly understood, especially in humans. Our findings call for studies investigating the mechanistic underpinnings of what is mediating stress-associated overeating of high sugar-foods, and how exactly this results in reduced HPA axis activation. 

## 5. Conclusions

This study provides novel supporting evidence in favor of a dampening effect on stress-induced cortisol responsiveness of real-world dietary intake of sugars. These observations add to a growing body of evidence reporting suppressive effects of high-energy foods on stress-associated glucocorticoid reactivity and are consistent with the comfort food hypothesis, where people are seen as motivated to eat palatable foods to alleviate the detrimental repercussions of stressor exposure.

We thus speculate that stress may be driving increasing consumption of calorically dense tasty foods, at least partially, because of these foods’ inhibitory effects on the stress-response hormonal network. This supposed advantage of seeking stress-relief in palatable food consumption carries significant implications for commonplace dietary recommendations imparted for ameliorating stress and stress-related pathologies. Importantly, however, while stress-induced eating may be an effective coping strategy in the short-term, habitual consumption of such foods promotes obesity, cardiovascular disease, and type 2 diabetes.

Given the pervasiveness of comfort eating in today’s world, its short-term benefits, and its downstream adverse long-term health effects, additional knowledge concerning the relation between stress and high-energy food consumption is required, especially considering that people are leading increasingly stressful lives while having more and more immediate and inexpensive access to palatable poorly nutritious foods. 

## Figures and Tables

**Table 1:** Descriptive statistics for Body Mass Index (BMI), Saturated Fats, Sugars, and the four cortisol measurement points.

	BMI	Saturated Fats	Sugars	Cortisol_0	Cortisol_20	Cortisol_35	Cortisol_65
	(kg/m^2^)	(% Daily Energy Intake)	(% Daily Energy Intake)	(µg/dL)	(µg/dL)	(µg/dL)	(µg/dL)
N	54	54	54	54	54	49	54
Missing	0	0	0	0	0	5	0
Mean	23.05	13.78	18.3	0.167	0.377	0.3	0.183
Standard Deviation	4.33	2.42	5.92	0.102	0.259	0.213	0.102
Minimum	16.76	8	8.93	0.012	0.097	0.074	0.052
Maximum	36.16	19	39.49	0.635	1.06	0.879	0.546

**Table 2:** Intercorrelations between covariates. Correlation Matrix.

	BMI	Saturated Fats	Sugars	Cortisol_0	Cortisol_20	Cortisol_35	Cortisol_65
	(kg/m^2^)	(% Daily Energy Intake)	(% Daily Energy Intake)	(µg/dL)	(µg/dL)	(µg/dL)	(µg/dL)
BMI	—						
Saturated Fats	−0.185	—					
Sugars	0.334 *	−0.259	—				
Cortisol_0	0.085	0.101	−0.014	—			
Cortisol_20	−0.28 *	−0.068	−0.366 **	0.12	—		
Cortisol_35	−0.211	−0.12	−0.307 *	0.01	0.823 ***	—	
Cortisol_65	−0.065	−0.023	−0.307 *	0.037	0.718 ***	0.865 ***	—

Note. * *p* < 0.05, ** *p* < 0.01, *** *p* < 0.001.

**Table 3:** Fixed effect parameter estimates for the final model.

Fixed Effects Parameter Estimates								
				95% CI				
Names	Effect	Estimate	SE	Lower	Upper	*df*	t	*p*
(Intercept)	(Intercept)	0.329	0.026	0.278	0.381	53.187	12.511	<0.001
Time	Time	0.082	0.016	0.051	0.113	56.381	5.13	<0.001
Gender	Female—Male	0.004	0.024	−0.044	0.051	90.293	0.156	0.876
Saturated Fats	Saturated Fats	−0.013	0.011	−0.035	0.009	51.64	−1.183	0.242
Sugars	Sugars	−0.014	0.005	−0.024	−0.005	54.298	−3.102	0.003
BMI	BMI	0.002	0.003	−0.003	0.008	90.846	0.724	0.471
Time^2^	Time^2^	−0.067	0.01	−0.087	−0.046	55.533	−6.45	<0.001
Time ∗ Saturated Fats	Time ∗ Saturated Fats	−0.01	0.007	−0.024	0.003	55.446	−1.494	0.141
Time ∗ Sugars	Time ∗ Sugars	−0.008	0.003	−0.013	−0.002	56.193	−2.776	0.007
Saturated Fats ∗ Time^2^	Saturated Fats ∗ Time^2^	0.006	0.004	−0.002	0.015	54.555	1.463	0.149
Sugars ∗ Time^2^	Sugars ∗ Time^2^	0.005	0.002	0.002	0.009	55.336	2.858	0.006

**Table 4:** Random components of the final model.

Random Components				
Groups	Name	*SD*	Variance	ICC
Participant	(Intercept)	0.178	0.032	0.756
	Time	0.093	0.009	
	Time^2^	0.063	0.004	
Residual		0.101	0.01	

Note. Number of Observations: 211, Groups: Participant, 54.

## References

[1] Sparling T.M., Cheng B., Deeney M., Santoso M.V., Pfeiffer E., Emerson J.A., Amadi F.M., Mitu K., Corvalan C., Verdeli H. (2021). Global Mental Health and Nutrition: Moving Toward a Convergent Research Agenda. Front. Public Health.

[2] Abbafati C., Abbas K.M., Abbasi-Kangevari M., Abd-Allah F., Abdelalim A., Abdollahi M., Abdollahpour I., Abegaz K.H., Abolhassani H., Aboyans V. (2020). Global Burden of 87 Risk Factors in 204 Countries and Territories, 1990–2019: A Systematic Analysis for the Global Burden of Disease Study 2019. Lancet.

[3] Mozaffarian D. (2020). Dietary and Policy Priorities to Reduce the Global Crises of Obesity and Diabetes. Nat. Food.

[4] Cordain L., Eaton S.B., Sebastian A., Mann N., Lindeberg S., Watkins B.A., O’Keefe J.H., Brand-Miller J. (2005). Origins and Evolution of the Western Diet: Health Implications for the 21st Century. Am. J. Clin. Nutr..

[5] Adan R.A.H., van der Beek E.M., Buitelaar J.K., Cryan J.F., Hebebrand J., Higgs S., Schellekens H., Dickson S.L. (2019). Nutritional Psychiatry: Towards Improving Mental Health by What You Eat. Eur. Neuropsychopharmacol..

[6] Lopresti A.L., Hood S.D., Drummond P.D. (2013). A Review of Lifestyle Factors That Contribute to Important Pathways Associated with Major Depression: Diet, Sleep and Exercise. J. Affect. Disord..

[7] Firth J., Siddiqi N., Koyanagi A., Siskind D., Rosenbaum S., Galletly C., Allan S., Caneo C., Carney R., Carvalho A.F. (2019). The Lancet Psychiatry Commission: A Blueprint for Protecting Physical Health in People with Mental Illness. Lancet Psychiatry.

[8] Firth J., Stubbs B., Teasdale S.B., Ward P.B., Veronese N., Shivappa N., Hebert J.R., Berk M., Yung A.R., Sarris J. (2018). Diet as a Hot Topic in Psychiatry: A Population-Scale Study of Nutritional Intake and Inflammatory Potential in Severe Mental Illness. World Psychiatry.

[9] Li Y., Lv M.R., Wei Y.J., Sun L., Zhang J.X., Zhang H.G., Li B. (2017). Dietary Patterns and Depression Risk: A Meta-Analysis. Psychiatry Res..

[10] Rahe C., Unrath M., Berger K. (2014). Dietary Patterns and the Risk of Depression in Adults: A Systematic Review of Observational Studies. Eur. J. Nutr..

[11] Marx W., Moseley G., Berk M., Jacka F. (2017). Nutritional Psychiatry: The Present State of the Evidence. Proc. Nutr. Soc..

[12] Jacka F.N. (2017). Nutritional Psychiatry: Where to Next?. EBioMedicine.

[13] Firth J., Marx W., Dash S., Carney R., Teasdale S.B., Solmi M., Stubbs B., Schuch F.B., Carvalho A.F., Jacka F. (2019). The Effects of Dietary Improvement on Symptoms of Depression and Anxiety: A Meta-Analysis of Randomized Controlled Trials. Psychosom. Med..

[14] Molendijk M., Molero P., Ortuño Sánchez-Pedreño F., Van der Does W., Angel Martínez-González M. (2018). Diet Quality and Depression Risk: A Systematic Review and Dose-Response Meta-Analysis of Prospective Studies. J. Affect. Disord..

[15] Malhi G.S., Bassett D., Boyce P., Bryant R., Fitzgerald P.B., Fritz K., Hopwood M., Lyndon B., Mulder R., Murray G. (2015). Royal Australian and New Zealand College of Psychiatrists Clinical Practice Guidelines for Mood Disorders. Aust. N. Z. J. Psychiatry.

[16] Andrews G., Bell C., Boyce P., Gale C., Lampe L., Marwat O., Rapee R., Wilkins G. (2018). Royal Australian and New Zealand College of Psychiatrists Clinical Practice Guidelines for the Treatment of Panic Disorder, Social Anxiety Disorder and Generalised Anxiety Disorder. Aust. N. Z. J. Psychiatry.

[17] Bremner J., Moazzami K., Wittbrodt M., Nye J., Lima B., Gillespie C., Rapaport M., Pearce B., Shah A., Vaccarino V. (2020). Diet, Stress and Mental Health. Nutrients.

[18] Arab A., Mehrabani S., Moradi S., Amani R. (2019). The Association between Diet and Mood: A Systematic Review of Current Literature. Psychiatry Res..

[19] Dionysopoulou S., Charmandari E., Bargiota A., Vlachos N., Mastorakos G., Valsamakis G. (2021). The Role of Hypothalamic Inflammation in Diet-Induced Obesity and Its Association with Cognitive and Mood Disorders. Nutrients.

[20] Morera L.P., Marchiori G.N., Medrano L.A., Defagó M.D. (2019). Stress, Dietary Patterns and Cardiovascular Disease: A Mini-Review. Front. Neurosci..

[21] Schweren L.J.S., Larsson H., Vinke P.C., Li L., Kvalvik L.G., Arias-Vasquez A., Haavik J., Hartman C.A. (2021). Diet Quality, Stress and Common Mental Health Problems: A Cohort Study of 121,008 Adults. Clin. Nutr..

[22] Herbison C.E., Allen K., Robinson M., Newnham J., Pennell C. (2017). The Impact of Life Stress on Adult Depression and Anxiety Is Dependent on Gender and Timing of Exposure. Dev. Psychopathol..

[23] Kendler K.S., Karkowski L.M., Prescott C.A. (1999). Causal Relationship between Stressful Life Events and the Onset of Major Depression. Am. J. Psychiatry.

[24] Plieger T., Melchers M., Montag C., Meermann R., Reuter M. (2015). Life Stress as Potential Risk Factor for Depression and Burnout. Burn. Res..

[25] Epel E., McEwen B., Seeman T., Matthews K., Castellazzo G., Brownell K.D., Bell J., Ickovics J.R. (2000). Stress and Body Shape: Stress-Induced Cortisol Secretion Is Consistently Greater among Women with Central Fat. Psychosom. Med..

[26] Epel E., Lapidus R., McEwen B., Brownell K. (2001). Stress May Add Bite to Appetite in Women: A Laboratory Study of Stress-Induced Cortisol and Eating Behavior. Psychoneuroendocrinology.

[27] Oliver G., Wardle J., Gibson E.L. (2000). Stress and Food Choice: A Laboratory Study. Psychosom. Med..

[28] Zellner D.A., Loaiza S., Gonzalez Z., Pita J., Morales J., Pecora D., Wolf A. (2006). Food Selection Changes under Stress. Physiol. Behav..

[29] Zellner D.A., Saito S., Gonzalez J. (2007). The Effect of Stress on Men’s Food Selection. Appetite.

[30] Adam T.C., Epel E.S. (2007). Stress, Eating and the Reward System. Physiol. Behav..

[31] Dallman M.F., Pecoraro N., Akana S.F., La Fleur S.E., Gomez F., Houshyar H., Bell M.E., Bhatnagar S., Laugero K.D., Manalo S. (2003). Chronic Stress and Obesity: A New View of “Comfort Food”. Proc. Natl. Acad. Sci. USA.

[32] Pecoraro N., Reyes F., Gomez F., Bhargava A., Dallman M.F. (2004). Chronic Stress Promotes Palatable Feeding, Which Reduces Signs of Stress: Feedforward and Feedback Effects of Chronic Stress. Endocrinology.

[33] Wilson M.E., Fisher J., Fischer A., Lee V., Harris R.B., Bartness T.J. (2008). Quantifying Food Intake in Socially Housed Monkeys: Social Status Effects on Caloric Consumption. Physiol. Behav..

[34] Arce M., Michopoulos V., Shepard K.N., Ha Q.C., Wilson M.E. (2010). Diet Choice, Cortisol Reactivity, and Emotional Feeding in Socially Housed Rhesus Monkeys. Physiol. Behav..

[35] Tomiyama J.A., Finch L.E., Cummings J.R. (2015). Did That Brownie Do Its Job? Stress, Eating, and the Biobehavioral Effects of Comfort Food. Emerging Trends in the Social and Behavioral Sciences.

[36] Finch L.E., Tomiyama A.J. (2014). Stress-Induced Eating Dampens Physiological and Behavioral Stress Responses. Nutrition in the Prevention and Treatment of Abdominal Obesity.

[37] Gonzalez M.J., Miranda-Massari J.R. (2014). Diet and Stress. Psychiatr. Clin. North Am..

[38] Singh K. (2016). Nutrient and Stress Management. J. Nutr. Food Sci..

[39] Appiakannan H.S., Rasimowicz M.L., Harrison C.B., Weber E.T. (2020). Differential Effects of High-Fat Diet on Glucose Tolerance, Food Intake, and Glucocorticoid Regulation in Male C57BL/6J and BALB/CJ Mice. Physiol. Behav..

[40] Tannenbaum B.M., Brindley D.N., Tannenbaum G.S., Dallman M.F., McArthur M.D., Meaney M.J. (1997). High-Fat Feeding Alters Both Basal and Stress-Induced Hypothalamic- Pituitary-Adrenal Activity in the Rat. Am. J. Physiol.-Endocrinol. Metab..

[41] Kamara K., Eskay R., Castonguay T. (1998). High-Fat Diets and Stress Responsivity. Physiol. Behav..

[42] Legendre A., Harris R.B.S. (2006). Exaggerated Response to Mild Stress in Rats Fed High-Fat Diet. Am. J. Physiol.-Regul. Integr. Comp. Physiol..

[43] von Dawans B., Zimmer P., Domes G. (2021). Effects of Glucose Intake on Stress Reactivity in Young, Healthy Men. Psychoneuroendocrinology.

[44] Zänkert S., Kudielka B.M., Wüst S. (2020). Effect of Sugar Administration on Cortisol Responses to Acute Psychosocial Stress. Psychoneuroendocrinology.

[45] Gonzalez-Bono E., Rohleder N., Hellhammer D.H., Salvador A., Kirschbaum C. (2002). Glucose but Not Protein or Fat Load Amplifies the Cortisol Response to Psychosocial Stress. Horm. Behav..

[46] Straznicky N.E., Louis W.J., McGrade P., Howes L.G. (1993). The Effects of Dietary Lipid Modification on Blood Pressure, Cardiovascular Reactivity and Sympathetic Activity in Man. J. Hypertens..

[47] Jakulj F., Zernicke K., Bacon S.L., Van Wielingen L.E., Key B.L., West S.G., Campbell T.S. (2007). A High-Fat Meal Increases Cardiovascular Reactivity to Psychological Stress in Healthy Young Adults. J. Nutr..

[48] La Fleur S.E., Houshyar H., Roy M., Dallman M.F. (2005). Choice of Lard, but Not Total Lard Calories, Damps Adrenocorticotropin Responses to Restraint. Endocrinology.

[49] Foster M.T., Warne J.P., Ginsberg A.B., Horneman H.F., Pecoraro N.C., Akana S.F., Dallman M.F. (2009). Palatable Foods, Stress, and Energy Stores Sculpt Corticotropin-Releasing Factor, Adrenocorticotropic and Corticosterone Concentrations after Restraint. Endocrinology.

[50] Tryon M.S., Stanhope K.L., Epel E.S., Mason A.E., Brown R., Medici V., Havel P.J., Laugero K.D. (2015). Excessive Sugar Consumption May Be a Difficult Habit to Break: A View from the Brain and Body. J. Clin. Endocrinol. Metab..

[51] Schwabe L., Haddad L., Schachinger H. (2008). HPA Axis Activation by a Socially Evaluated Cold-Pressor Test. Psychoneuroendocrinology.

[52] Chrousos G.P., Gold P.W. (1992). The Concepts of Stress and Stress System Disorders: Overview of Physical and Behavioral Homeostasis. JAMA J. Am. Med. Assoc..

[53] Zänkert S., Bellingrath S., Wüst S., Kudielka B.M. (2019). HPA Axis Responses to Psychological Challenge Linking Stress and Disease: What Do We Know on Sources of Intra- and Interindividual Variability?. Psychoneuroendocrinology.

[54] Flaa A., Ekeberg Ø., Kjeldsen S.E., Rostrup M. (2007). Personality May Influence Reactivity to Stress. Biopsychosoc. Med..

[55] Wu T., Snieder H., de Geus E. (2010). Genetic Influences on Cardiovascular Stress Reactivity. Neurosci. Biobehav. Rev..

[56] Stylianakis A.A. (2021). The Effect of Chronic Stress on the Adolescent Brain, Learning, and Memory. PhD Thesis.

[57] Lovibond S.H., Lovibond P.F. (1995). Manual for the Depression Anxiety Stress Scales.

[58] Aleknaviciute J., Tulen J.H.M., De Rijke Y.B., Bouwkamp C.G., van der Kroeg M., Timmermans M., Wester V.L., Bergink V., Hoogendijk W.J.G., Tiemeier H. (2017). The Levonorgestrel-Releasing Intrauterine Device Potentiates Stress Reactivity. Psychoneuroendocrinology.

[59] Hill E.E., Zack E., Battaglini C., Viru M., Viru A., Hackney A.C. (2008). Exercise and Circulating Cortisol Levels: The Intensity Threshold Effect. J. Endocrinol. Investig..

[60] Lovallo W.R., Whitsett T.L., Al’Absi M., Sung B.H., Vincent A.S., Wilson M.F. (2005). Caffeine Stimulation of Cortisol Secretion across the Waking Hours in Relation to Caffeine Intake Levels. Psychosom. Med..

[61] Stachowicz M., Lebiedzińska A. (2016). The Effect of Diet Components on the Level of Cortisol. Eur. Food Res. Technol..

[62] Watson J.F., Collins C.E., Sibbritt D.W., Dibley M.J., Garg M.L. (2009). Reproducibility and Comparative Validity of a Food Frequency Questionnaire for Australian Children and Adolescents. Int. J. Behav. Nutr. Phys. Act..

[63] Collins C.E., Burrows T.L., Truby H., Morgan P.J., Wright I.M.R., Davies P.S.W., Callister R. (2013). Comparison of Energy Intake in Toddlers Assessed by Food Frequency Questionnaire and Total Energy Expenditure Measured by the Doubly Labeled Water Method. J. Acad. Nutr. Diet..

[64] Collins C.E., Burrows T.L., Rollo M.E., Boggess M.M., Watson J.F., Guest M., Duncanson K., Pezdirc K., Hutchesson M.J. (2015). The Comparative Validity and Reproducibility of a Diet Quality Index for Adults: The Australian Recommended Food Score. Nutrients.

[65] Collins C.E., Boggess M.M., Watson J.F., Guest M., Duncanson K., Pezdirc K., Rollo M., Hutchesson M.J., Burrows T.L. (2014). Reproducibility and Comparative Validity of a Food Frequency Questionnaire for Australian Adults. Clin. Nutr..

[66] Marshall S., Watson J., Burrows T., Guest M., Collins C.E. (2012). The Development and Evaluation of the Australian Child and Adolescent Recommended Food Score: A Cross-Sectional Study. Nutr. J..

[67] Goldfein K.R., Slavin J.L. (2015). Why Sugar Is Added to Food: Food Science 101. Compr. Rev. Food Sci. Food Saf..

[68] Hess J., Latulippe M.E., Ayoob K., Slavin J. (2012). The Confusing World of Dietary Sugars: Definitions, Intakes, Food Sources and International Dietary Recommendations. Food Funct..

[69] Sjörs A., Ljung T., Jonsdottir I.H. (2014). Diurnal Salivary Cortisol in Relation to Perceived Stress at Home and at Work in Healthy Men and Women. Biol. Psychol..

[70] Gallucci M. (2019). GAMLj: General Analyses for Linear Models. *Jamovi Modul*. https://gamlj.github.io/.

[71] The Jamovi Project. *Jamovi* (Version 1.8.1). 2021. [Computer Software]. https://www.jamovi.org/.

[72] R Core Team (2021). R: A Language and Environment for Statistical Computing.

[73] Satterthwaite F.E. (1946). An Approximate Distribution of Estimates of Variance Components. Biom. Bull..

[74] Gelman A., Hill J. (2006). Data Analysis Using Regression and Multilevel/Hierarchical Models..

[75] Goldstein H. (1986). Multilevel Mixed Linear Model Analysis Using Iterative Generalized Least Squares. Biometrika.

[76] Bryk A.S., Raudenbush S.W. (1987). Application of Hierarchical Linear Models to Assessing Change. Psychol. Bull..

[77] Singer J.D., Willet J.B. (2003). Applied Longitudinal Data Analysis: Modeling Change and Event Occurrence.

[78] Snijders T.A.B., Bosker R. (1999). Multilevel Analysis: An Introduction to Basic and Advanced Multilevel Modeling.

[79] Mathieu J.E., Aguinis H., Culpepper S.A., Chen G. (2012). Understanding and Estimating the Power to Detect Cross-Level Interaction Effects in Multilevel Modeling. J. Appl. Psychol..

[80] Nezlek J.B., Mroziński B. (2020). Applications of Multilevel Modeling in Psychological Science: Intensive Repeated Measures Designs. L’Année Psychol..

[81] Verma R., Balhara Y.S., Gupta C. (2012). Gender Differences in Stress Response: Role of Developmental and Biological Determinants. Ind. Psychiatry J..

[82] Bryant R.A., McGrath C., Felmingham K.L. (2013). The Roles of Noradrenergic and Glucocorticoid Activation in the Development of Intrusive Memories. PLoS ONE.

[83] Gutiérrez-Pliego L.E., Del Socorro Camarillo-Romero E., Montenegro-Morales L.P., De Jesus Garduño-García J. (2016). Dietary Patterns Associated with Body Mass Index (BMI) and Lifestyle in Mexican Adolescents. BMC Public Health.

[84] Newby P.K., Muller D., Hallfrisch J., Qiao N., Andres R., Tucker K.L. (2003). Dietary Patterns and Changes in Body Mass Index and Waist Circumference in Adults. Am. J. Clin. Nutr..

[85] Majeed F. (2015). Association of BMI with Diet and Physical Activity of Female Medical Students at the University of Dammam, Kingdom of Saudi Arabia. J. Taibah Univ. Med. Sci..

[86] Browne W.J., Draper D. (2006). A Comparison of Bayesian and Likelihood-Based Methods for Fitting Multilevel Models. Bayesian Anal..

[87] Maas C.J.M., Hox J.J. (2005). Sufficient Sample Sizes for Multilevel Modeling. Methodology.

[88] McNeish D., Stapleton L.M. (2016). The Effect of Small Sample Size on Two-Level Model Estimates: A Review and Illustration. Educ. Psychol. Rev..

[89] McNeish D. (2017). Small Sample Methods for Multilevel Modeling: A Colloquial Elucidation of REML and the Kenward-Roger Correction. Multivar. Behav. Res..

[90] Biesanz J.C., Deeb-Sossa N., Papadakis A.A., Bollen K.A., Curran P.J. (2004). The Role of Coding Time in Estimating and Interpreting Growth Curve Models. Psychol. Methods.

[91] King K.M., Littlefield A.K., McCabe C.J., Mills K.L., Flournoy J., Chassin L. (2018). Longitudinal Modeling in Developmental Neuroimaging Research: Common Challenges, and Solutions from Developmental Psychology. Dev. Cogn. Neurosci..

[92] Byrk A.S., Raudenbush S.W., Congdon R. (2008). HLM 7 for Windows.

[93] Cohen J. (1978). Partialed Products Are Interactions; Partialed Powers Are Curve Components. Psychol. Bull..

[94] Aiken L., West S. (1991). Multiple Regression: Testing and Interpreting Interactions.

[95] West S.G., Finch J.F., Curran P.J., Hoyle R.H. (1995). Structural Equation Models with Nonnormal Variables: Problems and Remedies. Structural Equation Modeling: Concepts, Issues, and Applications.

[96] Kim H.-Y. (2013). Statistical Notes for Clinical Researchers: Assessing Normal Distribution (2) Using Skewness and Kurtosis. Restor. Dent. Endod..

[97] Kline R.B. (2004). Principles and Practice of Structural Equation Modeling.

[98] World Health Organization (2015). Guideline: Sugars Intake for Adults and Children.

[99] Drewnowski A., Rehm C.D. (2014). Consumption of Added Sugars among Us Children and Adults by Food Purchase Location and Food Source. Am. J. Clin. Nutr..

[100] Ulrich-Lai Y.M., Christiansen A.M., Ostrander M.M., Jones A.A., Jones K.R., Choi D.C., Krause E.G., Evanson N.K., Furay A.R., Davis J.F. (2010). Pleasurable Behaviors Reduce Stress via Brain Reward Pathways. Proc. Natl. Acad. Sci. USA.

[101] Laugero K., Bell M.E., Bhatnagar S., Soriano L., Dallman M.F. (2001). Sucrose Ingestion Normalizes Central Expression of Corticotropin-Releasing-Factor Messenger Ribonucleic Acid and Energy Balance in Adrenalectomized Rats: A Glucocorticoid-Metabolic-Brain Axis?. Endocrinology.

[102] Laugero K., Gomez F., Manalo S., Dallman M.F. (2002). Corticosterone Infused Intracerebroventricularly Inhibits Energy Storage and Stimulates the Hypothalamo-Pituitary Axis in Adrenalectomized Rats Drinking Sucrose. Endocrinology.

[103] Markus R., Panhuysen G., Tuiten A., Koppeschaar H. (2000). Effects of Food on Cortisol and Mood in Vulnerable Subjects under Controllable and Uncontrollable Stress. Physiol. Behav..

[104] Warne J.P. (2009). Shaping the Stress Response: Interplay of Palatable Food Choices, Glucocorticoids, Insulin and Abdominal Obesity. Mol. Cell. Endocrinol..

[105] Oliver G., Wardle J. (1999). Perceived Effects of Stress on Food Choice. Physiol. Behav..

[106] Rutters F., Nieuwenhuizen A.G., Lemmens S.G.T., Born J.M., Westerterp-Plantenga M.S. (2009). Acute Stress-Related Changes in Eating in the Absence of Hunger. Obesity.

[107] Kim Y., Yang H.Y., Kim A.J., Lim Y. (2013). Academic Stress Levels Were Positively Associated with Sweet Food Consumption among Korean High-School Students. Nutrition.

[108] Polivy J., Herman C.P., McFarlane T. (1994). Effects of Anxiety on Eating: Does Palatability Moderate Distress-Induced Overeating in Dieters?. J. Abnorm. Psychol..

[109] Leigh Gibson E. (2006). Emotional Influences on Food Choice: Sensory, Physiological and Psychological Pathways. Physiol. Behav..

[110] Macht M., Mueller J. (2007). Immediate Effects of Chocolate on Experimentally Induced Mood States. Appetite.

[111] Ulrich-Lai Y.M. (2016). Self-Medication with Sucrose. Curr. Opin. Behav. Sci..

[112] Bell M.E., Bhatnagar S., Liang J., Soriano L., Nagy T.R., Dallman M.F. (2000). Voluntary Sucrose Ingestion, like Corticosterone Replacement, Prevents the Metabolic Deficits of Adrenalectomy. J. Neuroendocrinol..

[113] Dallman M.F., Pecoraro N.C., La Fleur S.E., Warne J.P., Ginsberg A.B., Akana S.F., Laugero K.C., Houshyar H., Strack A.M., Bhatnagar S. (2006). Glucocorticoids, Chronic Stress, and Obesity. Prog. Brain Res..

[114] Dallman M.F., Akana S.F., Strack A.M., Scribner K.S., Pecoraro N., La Fleur S.E., Houshyar H., Gomez F. (2004). Chronic Stress-Induced Effects of Corticosterone on Brain: Direct and Indirect. Ann. N. Y. Acad. Sci..

[115] Peters A., Schweiger U., Pellerin L., Hubold C., Oltmanns K.M., Conrad M., Schultes B., Born J., Fehm H.L. (2004). The Selfish Brain: Competition for Energy Resources. Neurosci. Biobehav. Rev..

[116] Ulrich-Lai Y.M., Ostrander M.M., Thomas I.M., Packard B.A., Furay A.R., Dolgas C.M., Van Hooren D.C., Figueiredo H.F., Mueller N.K., Choi D.C. (2007). Daily Limited Access to Sweetened Drink Attenuates Hypothalamic-Pituitary- Adrenocortical Axis Stress Responses. Endocrinology.

[117] Drolet G., Dumont E.C., Gosselin I., Kinkead R., Laforest S., Trottier J.F. (2001). Role of Endogenous Opioid System in the Regulation of the Stress Response. Prog. Neuro-Psychopharmacol. Biol. Psychiatry.

[118] Daubenmier J., Lustig R.H., Hecht F.M., Kristeller J., Woolley J., Adam T., Dallman M., Epel E. (2014). A New Biomarker of Hedonic Eating? A Preliminary Investigation of Cortisol and Nausea Responses to Acute Opioid Blockade. Appetite.

[119] McEwen B.S. (2013). Erratum: Brain on Stress: How the Social Environment Gets under the Skin. Proc. Natl. Acad. Sci. USA.

[120] Pruessner J.C., Dedovic K., Khalili-Mahani N., Engert V., Pruessner M., Buss C., Renwick R., Dagher A., Meaney M.J., Lupien S. (2008). Deactivation of the Limbic System During Acute Psychosocial Stress: Evidence from Positron Emission Tomography and Functional Magnetic Resonance Imaging Studies. Biol. Psychiatry.

[121] Daubenmier J., Kristeller J., Hecht F.M., Maninger N., Kuwata M., Jhaveri K., Lustig R.H., Kemeny M., Karan L., Epel E. (2011). Mindfulness Intervention for Stress Eating to Reduce Cortisol and Abdominal Fat among Overweight and Obese Women: An Exploratory Randomized Controlled Study. J. Obes..

[122] Dallman M.F., Pecoraro N.C., La Fleur S.E. (2005). Chronic Stress and Comfort Foods: Self-Medication and Abdominal Obesity. Brain. Behav. Immun..

[123] Friedman J.M. (2003). A War on Obesity, Not the Obese. Science.

[124] Stunkard A.J., Faith M.S., Allison K.C. (2003). Depression and Obesity. Biol. Psychiatry.

[125] Bjorntorp P. (1990). “Portal” Adipose Tissue as a Generator of Risk Factors for Cardiovascular Disease and Diabetes. Arteriosclerosis.

[126] Wajchenberg B.L., Lé B., Wajchenberg O. (2000). Subcutaneous and Visceral Adipose Tissue. Endocr. Rev..

[127] Maniam J., Morris M.J. (2010). Palatable Cafeteria Diet Ameliorates Anxiety and Depression-like Symptoms Following an Adverse Early Environment. Psychoneuroendocrinology.

[128] Konttinen H., Männistö S., Sarlio-Lähteenkorva S., Silventoinen K., Haukkala A. (2010). Emotional Eating, Depressive Symptoms and Self-Reported Food Consumption. A Population-Based Study. Appetite.

[129] Schuman M., Gitlin M.J., Fairbanks L. (1987). Sweets, Chocolate, and Atypical Depressive Traits. J. Nerv. Ment. Dis..

[130] Parker G., Roy K., Mitchell P., Wilhelm K., Malhi G., Hadzi-Pavlovic D. (2002). Atypical Depression: A Reappraisal. Am. J. Psychiatry.

[131] Collins S., Dash S., Allender S., Jacka F., Hoare E. (2022). Diet and Mental Health During Emerging Adulthood: A Systematic Review. Emerg. Adulthood.

